# DASUNet: a deeply supervised change detection network integrating full-scale features

**DOI:** 10.1038/s41598-024-63257-8

**Published:** 2024-05-30

**Authors:** Ru Miao, Geng Meng, Ke Zhou, Yi Li, Ranran Chang, Guangyu Zhang

**Affiliations:** 1 School of Computer and Information Engineering, Henan University, Kaifeng, 475004 People’s Republic of China; 2 Henan Province Engineering Research Center of Spatial Information Processing, Henan University, Kaifeng, 475004 People’s Republic of China; 3 Henan Provincial Spatio-Temporal Big Data Technology Innovation Center, Zhengzhou, 450046 People’s Republic of China

**Keywords:** Change detection, Atrous spatial pyramid pooling, Full-scale feature fusion, Deeply supervised layers, Computer science, Environmental impact, Computational science

## Abstract

The change detection (CD) technology has greatly improved the ability to interpret land surface changes. Deep learning (DL) methods have been widely used in the field of CD due to its high detection accuracy and application range. DL-based CD methods usually cannot fuse the extracted feature information at full scale, leaving out effective information, and commonly use transfer learning methods, which rely on the original dataset and training weights. To address the above issues, we propose a deeply supervised (DS) change detection network (DASUNet) that fuses full-scale features, which adopts a Siamese architecture, fuses full-scale feature information, and realizes end-to-end training. In order to obtain higher feature information, the network uses atrous spatial pyramid pooling (ASPP) module in the coding stage. In addition, the DS module is used in the decoding stage to exploit feature information at each scale in the final prediction. The experimental comparison shows that the proposed network has the current state-of-the-art performance on the CDD and the WHU-CD, reaching 94.32% and 90.37% on F1, respectively.

## Introduction

In practical applications, change detection (CD) is to identify differences in different time-phase remote sensing images in the same area. At present, with the advancement of high-resolution remote sensing satellite processing and application technology, a large amount of remote sensing image data has emerged, with larger coverage and finer display accuracy. By analyzing remote sensing images of different phases, CD can judge the change characteristics of the same area with less labor cost and higher accuracy, and identify them, so as to provide decision support for land protection and utilization, disaster monitoring, urban planning, etc.

Traditional CD methods can generally be divided into: (1) pixel-based methods and (2) object-based methods. In pixel-based methods, arithmetic operations are usually used to compare the pixel values of a two-phase image, such as image differences^1^, Image regression^2^ and image ratios^3^. Then, according to the threshold, the image pixels are divided into variation or non-variation classes, which mainly focus on spectral values and mostly ignore spatial context information^4^. Based on Bayesian theory, Bruzzone et al. proposed two image difference recognition techniques^5^. Zerrouki et al. combines a multivariate exponential weighted moving average (MEWMA) plot with a support vector machine (SVM) to detect changes in the land surface^6^. In the object-based approach, object features are usually established based on the spectra, texture, geometry and other information in the image, such as change vector analysis (CVA)^4^, multivariate alteration detection (MAD)^7^ and principal component analysis (PCA)^8^, and so on. Although this kind of method takes into account spatial context information, artificial feature extraction is complex^9^. Based on multi-scale uncertainty analysis, Zhang et al. proposed a new object-based change detection technology^10^. Wu et al. designed a post-classification method based on Bayesian soft fusion and Iterative Slow Feature Analysis (ISFA)^11^.

Since 2012, deep learning technologies have demonstrated significant potential in the image detection and classification. Deep neural networks are particularly suitable for processing detailed feature in high-resolution images, so CD networks are generally closer to deep learning. In the CD networks using deep learning, the pixel-based method is difficult to fully utilize the image spatial information, and the object-based method is limited by the uncertainty of segmenting the object^12^, while the method based on depth features directly learns end-to-end from the labeled change map, which effectively overcomes the influence of light intensity, seasonal change and other factors, and shows good performance^13^. At present, CD methods are mainly based on the extraction of deep features, which utilizes a fully convolutional deep neural network (FCN) to convert the bitemporal images into a high-dimensional space, then uses the depth features as an analysis unit to generate the final change map^13–17^. The deep feature methods can be further divided into early fusion (EF) and Siamese architectures according to the single-flow structure and dual-flow structure. Daudt et al. first proposed these two architectures and applied them to urban multispectral image CD, and later fused fully convolutional neural network to propose an early fusion and Siamese architecture based on UNet, which used the end-to-end approach to realize the semantic-level segmentation of bitemporal images^13,17^. In the early stage of fusion, bitemporal images were fed into the neural network after combining along the channel dimension, because the network of semantic segmentation of a single image is often used, which is prone to missed detection or false detection in large areas. Peng et al. used EF for UNet++, and concatenated different hierarchical change diagrams of the multi-sided output^18^. The Siamese architecture generally uses a network with shared weights to extract the depth features of bitemporal images. Daudt et al. compared the Siamese architecture with the early fusion, and the results showed that the Siamese architecture retains more features of the position information of the bitemporal images, and the detection accuracy is greatly improved^13^. Based on Siamese architecture, Chen et al. designed a spatiotemporal attention module using the self-attention mechanism, and divided the image into multiple scale subregions, which can obtain spatiotemporal correlation at different scales^19^. Lei et al. proposed a pseudo-Siamese structure, which extracts features by a dual-stream structure, but the weights are not shared^20^. Shi et al. adopt Siamese architecture, and proposed a new network based on deeply supervised attention measurement^21^. Zhang et al. used a Siamese architecture to design a deeply supervised network that fuses channels and spatial attention^12^. The success of transformer in natural language processing (NLP) has led researchers to apply it to a variety of computer vision tasks, and Siamese change detection methods using transformer to process features have emerged. Bandara et al. proposed a transformer-based end-to-end Siamese network architecture for change detection^22^. Based on the Siamese architecture, Chen et al. proposed a bitemporal image transformer to efficiently and effectively model contexts within the spatial–temporal domain^23^.

The existing deep learning CD networks often draws on the semantic segmentation networks of a single image. The skip connections structure in semantic segmentation can combine low-level detail information with high-level semantic information, so that the prediction region boundary and shape information obtained are more accurate^13,24,25^. Among them, the UNet series has achieved good detection results with its unique skip connections structure, so it is widely used in the field of CD^24,26,27^. Daudt et al. proposed the early fusion and the Siamese architecture based on UNet^13^. Codegoni et al. designed a Siamese UNet backbone network for feature extraction by drawing on the UNet structure^28^. Fang et al. used the Siamese structure for UNet++ and designed a densely connected Siamese network for CD^29^. The application of transformer in semantic segmentation also draws on the UNet series. Based on the Swin transformer, Cao et al. proposed a UNet-like pure Transformer network, which is used for medical image segmentation^30^. Chen et al. combined UNet++ with Swin Transformer to propose an automatic medical image segmentation method^31^. The above semantic segmentation model can be modified for change detection, and there are also change detection methods based on transformer. Tang et al. combined Swin Transformer, UNet and Siamese architecture to design a network for remote sensing image change detection^32^. To solve the problem of the quality of feature differences, Guo et al. proposed iterative difference-enhanced transformers (IDET) to optimize feature differences^33^.

However, existing CD networks still have some problems. First of all, previous studies did not fully utilize the multi-scale features extracted in the feature fusion stage, and often only used the features of two adjacent scales. Therefore, in the subsequent prediction, areas of change may be missed in terms of location and shape. Secondly, the information extracted by the hidden layers are not fully utilized, which can significantly affect the subsequent prediction, resulting in insufficient boundary or shape detection of the change area. In addition, transformer has the problems of low computational efficiency and lack of space limitation in the field of computer vision^34–36^, and at the same time, compared with convolutional neural networks (CNNs), this architecture lacks advantage in parameter sharing and dealing with the problem of bitemporal images change detection^20,28^. Finally, in order to speed up the training, many methods use transfer learning, but ignore the differences between the trained dataset and the change detection dataset, which affects the final detection effect.

To address the above issues, a deeply supervised change detection network integrating full-scale features is proposed. Firstly, based on CNNs, this network uses the Siamese structure to extract bitemporal features, receives full-scale feature information in the decoding stage, fuses global-scale features, and realizes end-to-end training. Secondly, the network uses the ASPP module in the coding stage^37^, fused with multi-scale convolutional kernels, and obtained higher-level feature representations. To accelerate model convergence, a deep supervision mechanism is used in the decoding stage to fully leverage the role of feature at each scale in the final prediction.

The main contributions of this article are as follows:

1. A full-scale skip connections structure is proposed for CD networks, which allows each decoder layer to combine the larger scale feature maps from the decoder and the smaller and same-scale feature maps from the encoder to obtain richer feature information.
2. We propose a new CD network, DASUNet, which integrates ASPP module into the encoder layer and uses the DS layer to obtain more discriminative features.
3. The proposed DASUNet achieves state-of-the-art (SOTA) performance on the CDD benchmark dataset and the WHU-CD building dataset, with F1 scores of up to 94.32% and 90.37%, respectively.

The structure of this paper is shown below: “Materials and methods” provides the proposed networks, while “Results” presents the setup and results of all experiments. The Discussion is presented in “Discussion”. In “Conclusions”, we summarized the article.

## Materials and methods

This section, we describe the network model DASUNet in detail. First, we briefly describe how the various parts of the DASUNet network work. Then, the main structures designed in the network will be detailed, including the full-scale skip connection structure in CD, ASPP, and deep supervision. Finally, we will introduce the loss function, which is closely related to deep supervision.

### The proposed DASUNet network

**Figure 1:**
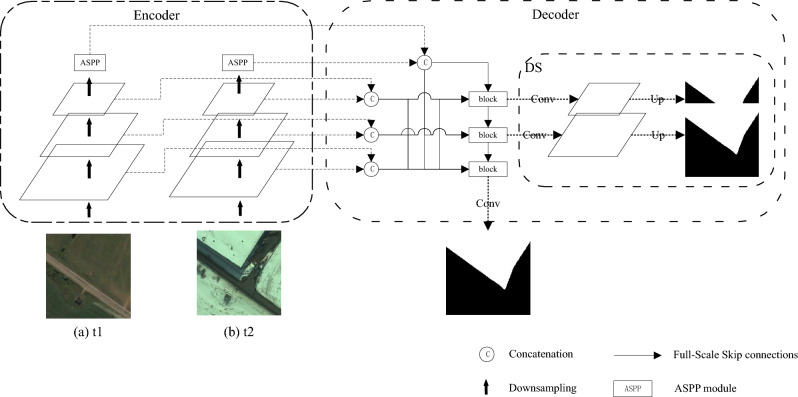
Overview of the DASUNet^38^. (**a**) The first phase image t1, (**b**) the second phase image t2.

In the section, we provide a brief overview of the proposed DASUNet. Figure 1 shows the architecture of this network. It comprises an encoding stage, a decoding stage, and a DS module. In the encoding stage, the encoders that share weights extract the features of the bitemporal images separately, and then in this stage, the ASPP module is used to extract higher-level feature representations. After that, the bitemporal features extracted from each encoder layer are concatenated. During the decoding phase, the concatenative information is passed to the decoder layer via a full-size skip connection. Finally, deep supervision is used to learn for each encoder layer.

### Full-scale skip connection structure in CD

In the field of CD, the objects to be detected are often complex and diverse, ranging from buildings to automobiles, and vary in size. In feature information fusion, the decoder layer of the previous network usually only uses the feature information of adjacent scales, and does not fully utilize the feature information of the whole scale, resulting in the loss of small targets or abnormal target positions.

In the decoding stage of this article, full-scale skip connections are adopted, which can combine low-level details and high-level semantics from feature maps at different scales. In order to accurately identify changing objects, both accurate high-level semantic information and position information are required, and full-scale skip connections can send the information to each decoder layer to fuse global features at each scale.

**Figure 2:**
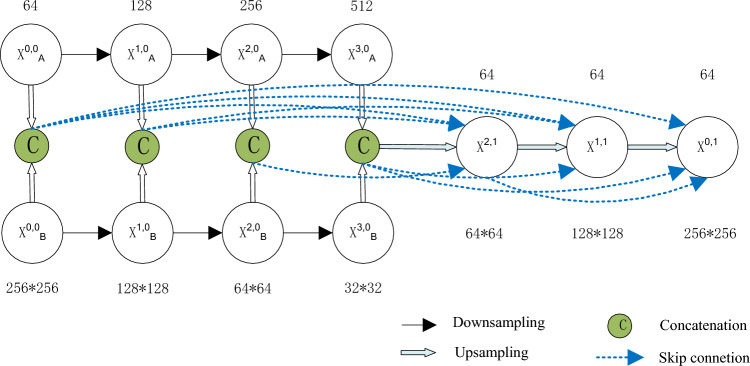
Full-scale skip connections in CD.

In Fig. 2, the subscript of X is divided into A and B, where A represents the first phase encoder and B represents the second phase encoder. An X with a superscript (x, 0) indicates the encoder, where x is 0, 1, 2, 3, representing encoders of different scales. The number of channels for the encoder to extract features is 64, 128, 256, and 512, and the width and height are 256 × 256, 128 × 128, 64 × 64, 32 × 32, respectively. An X with a superscript (x, 1) represents the decoder, where x is 2, 1, 0, which represents the full-scale feature convolution block that receives the extraction. The number of channels for the decoder to extract features is 64, 64, and 64, respectively, and the width and height are 64 × 64, 128 × 128, 256 × 256, respectively.

Compared with the semantic segmentation of a single image, change detection places greater emphasis on the matching of bitemporal feature maps. In view of the particularity of CD, the full-scale skip connections in this article no longer performs the channel alignment operation of each scale feature map.

Taking the decoder X^1,1^ as an example, you need to accept the bitemporal features extracted by the X^3,0^, X^1,0^, X^0,0^ encoder and high semantic features after decoder X^2,1^ processing. Let x^1,1^ represents the output of X^1,1^, and X^(x,0)^ is the bitemporal features extracted by the X^(x,0)^ encoder. The stack of feature maps represented by x^1,1^ is computed as:

1 $${x}^{\text{1,1}}=h\left(\left[u\left({x}^{\text{3,0}}\right),{u\left({x}^{\text{2,1}}\right),x}^{\text{1,0}},p\left({x}^{\text{0,0}}\right)\right]\right)$$

where h(∙) represents the convolutional block operation, [∙] represents the concatenation, u(∙) indicates an up-sampling operation, and p(∙) indicates a down-sampling operation.

The encoder layer is indexed with i, and x^(i,0)^ represent the two-phase features extracted by the encoder layer X^(i,0)^. The decoder layer is indexed with j to represent the high semantic features generated by the decoder layer. The decoder output can be expressed as:

2 $${x}^{j,1}=\left\{\begin{array}{c}h\left(\left[p\left({x}_{i<j}^{i,0}\right),{x}_{i=j}^{j,0},u\left({x}_{i>j,i<3}^{i,1}\right),u\left({x}_{i=3}^{3,0}\right)\right]\right), \quad j<2;i=\text{0,1},\text{2,3}\\ h\left(\left[p\left({x}_{i<j}^{i,0}\right),{x}_{i=j}^{i,0},u\left({x}_{i>j}^{i,0}\right)\right]\right), \quad j=2;i=\text{0,1},\text{2,3}\end{array}\right.$$

where h(∙) represents the convolutional block operation, [∙] represents the concatenation, u(∙) indicates an up-sampling operation, and p(∙) indicates a down-sampling operation.

**Figure 3:**
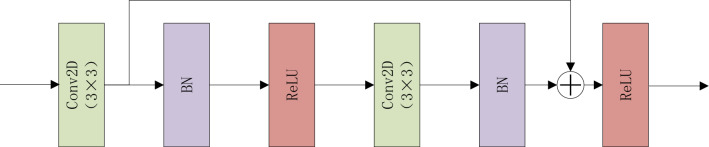
The convolutional blocks.

In this article, the convolution block adopts a residual structure (Fig. 3), and the residual connection line is placed after the first convolutional layer, and an additional 1 × 1 convolutional layer is no longer required for the channel number transformation. On the one hand, this design reduces the number of parameters compared with the traditional residual convolutional block design. On the other hand, the 3 × 3 convolutional layer has a larger receptive field than the 1 × 1 convolutional layer, extracts more abundant feature information, and has more advantages in the identity mapping of the residual structure.

### ASPP module

**Figure 4:**
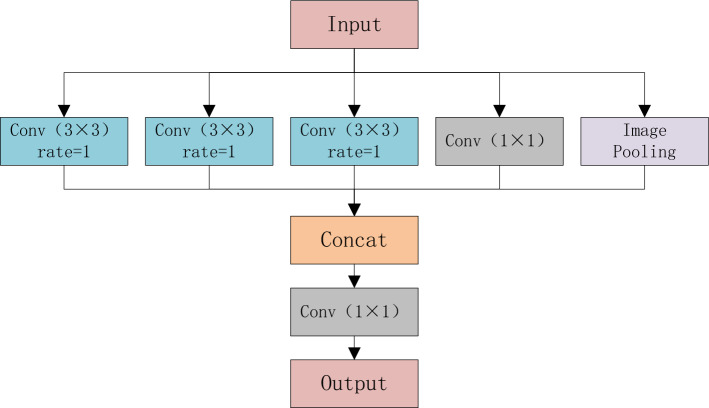
The ASPP module.

High-resolution images contain rich information, and the detection targets in the images are often more complex and diverse. Therefore, in this article, the original image is down-sampled by a factor of eight instead of sixteen to preserve more of the original information. The traditional convolutional block uses 3 × 3 convolutional kernels, and the field of view is very small and is difficult to distinguish between pairs of features that represent non-obvious. In this article, the ASPP module (Fig. 4) is used to expand the convolution field by using dilated convolution, and the spatial pyramid structure is utilized to obtain rich feature information.

Specifically, the ASPP module divides the input into five pathways: three atrous convolutions, with kernel sizes of 3 × 3 and atrous rates of 1, 2, and 3, respectively, which are mainly used to expand the receptive field and extract richer feature information; a 1 × 1 convolution for dimensionality reduction; Image Pooling is used to complement global features. Finally, the output of these five layers is concatenated, and the dimensionality is reduced to a given number of channels with a 1 × 1 convolutional layer. Let x^in^ and x^out^ represent the input and output features, respectively, and the ASPP module can be represented as follows:

3 $${x}^{out}=C\left(\left[{AC}^{1}\left({x}^{in}\right),{AC}^{2}\left({x}^{in}\right),{AC}^{3}\left({x}^{in}\right),C\left({x}^{in}\right),U\left(C\left(P\left({x}^{in}\right)\right)\right)\right]\right)$$

where C(∙) stands for the convolutional block. AC(∙) stands for the atrous convolutional block, and both padding and dilation rates are determined by superscript. [∙] represents the concatenation, U(∙) indicates an up-sampling operation, and P(∙) indicates a down-sampling operation.

Considering that the ASPP module in this article is located in the last layer of the encoding stage, and the original image has been pooled for multiple rounds, the void rate of the void convolution is set to 1, 2, 3.

### DS module

In general, most traditional end-to-end deep convolutional neural networks only provide supervision of the output layer. However, the training of the hidden layer of deep convolutional networks is unsupervised, which will inevitably affect the subsequent prediction.

Therefore, this article uses a DS module to supervise all three decoder layers, which helps the hidden layers learn more discriminative features to improve the prediction accuracy.

As an example, we can express the weights of each layer from input to output as W ^(1)^, …, W^(n)^ for a common end-to-end convolutional network of N layers. And the weight of the output layer is W^(n)^. The weights of the output layer and all previous layers are recorded as W^n^ = {W^(1)^, …, W^(n)^}, and the objective function can be computed as:

4 $$P\left(W\right)=L\left({W}^{n},T\right)$$

where T represents the true label, and L (W^n^, T) is the loss directly determined by W^n^.

The outputs of the two additional hidden layers and the final layer this article are represented as out-1, out-2 and out-3. We can express the weight of each layer from input to output as W ^(1)^, …, W^(out-1)^, …, W^(out-2)^, …, W ^(out-3)^, where the weight of the output layers are W^(out-1)^, W^(out-2)^ and W^(out-3)^, respectively. Denote the weights of the three output layers and all previous layers as W^out-1^ = {W^(1)^,…,W^(out-1)^}, W^out-2^ = {W^(1)^,…,W^(out-1)^,…,W^(out-2)^} and W^out-3^ = {W^(1)^,…,W^(out-1)^,…,W^(out-2)^,…,W^(out-3)^}. The objective function in this article can be computed as:

5 $$P\left(W\right)=\sum_{m=1}^{3}{a}_{m}L\left({W}^{out-m},T\right)$$

where m represents the output layer index, and a is the weight factor of the corresponding output layer in the total loss function.

It is worth mentioning that, the outputs of the two additional hidden layers are fed into a 1 × 1 convolutional layer, and then restored to the original image size through bilinear up-sampling.

### Loss function

In this article, to weaken the influence of positive and negative sample imbalance, the loss function combines cross-entropy (ce) loss and dice loss (dice). The formula for the composite loss function is computed as:

6 $$L={L}_{ce}+{L}_{dice}.$$

The cross-entropy loss formula is computed as:

7 $${L}_{ce}=\frac{1}{N}\sum_{n=1}^{N}-{Y}_{n}\text{log}\left({P}_{n}\right)-\left(1-{Y}_{n}\right)\text{log}(1-{P}_{n}).$$

The formula for the loss of the dice coefficient is as follows:

8 $${L}_{dice}=1-\frac{2\sum_{n=1}^{N}{Y}_{n}{P}_{n}}{\sum_{n=1}^{N}{Y}_{n}+\sum_{n=1}^{N}{P}_{n}}.$$

where N is the number of pixels, Y_n_ is the true value of the category, and P_n_ is the predicted value of the model.

In this article, the deep supervision mechanism is adopted, the weight value of each side output is set to 1, and the loss function of the model can be calculated as:

9 $$L=\sum_{n=1}^{3}{L}^{n}.$$

where $${L}^{n}={L}_{ce}^{n}+{L}_{dice}^{n}$$.

## Results

### Experimental setup

In this section, experimental environment, experimental datasets and corresponding evaluation indicators are described in detail. Then we conducted experiments on CDD and WHU-CD datasets to verify the model effectiveness. The advantages of this model are pointed out by comparing the model with similar models, and then the contribution of each submodule is verified by ablation experiments.

#### Experimental environment

In this experiment, the model iteration is set to 100 times, the initial learning rate is 0.001. The learning rate is updated by using a fixed-length decay strategy, and the learning rate is halved every 6 epochs, and the batch size is set to 8. AdamW was used to optimize the model parameters.

To increase the diversity of data, the training dataset is enhanced during training, including vertical and horizontal flipping, and random 90-degree, 180-degree, and 270-degree rotation of the image. All methods are implemented based on the Pytorch framework, and the hardware environment is NVIDIA Tesla-T4 16 GB GPU.

#### Datasets

The CDD dataset is a public seasonal CD dataset. The dataset contains 11 pairs of seasonal change images, four pairs of sizes 1900 × 1000 and seven are 4725 × 2200. The spatial resolution of the image is 3–100 cm/px^39^. The image is cropped into sub-images of a size of 256 × 256. The final dataset contains 16,000 image pairs, which are divided into a training set, a test set, and a validation set according to 10:3:3.

WHU-CD is a public building CD dataset^40^. The original dataset contains two datasets, in which the training set contains a pair of aerial images of 21,243 × 15,354 in 2012 and 2018, and the test set contains a pair of aerial images of 11,265 × 15,354 of the same age, all with a spatial resolution of 0.075 m. According to the dataset division standard, the fused aerial images of 32,507 × 15,354 are cropped into blocks of 256 × 256 size, and there was no overlap. Then the whole images were randomly divided into 5204 pairs of training set, 744 pairs of validation set and 1486 pairs of testing set according to the ratio of 7:1:2.

#### Evaluation metrics

In this article, we used four indicators to evaluate the model performance on the CDD dataset and WHU-CD dataset, namely: accuracy (OA), precision (P), recall (R), F1score (F1). These metrics are defined as:

10 $$OA=\frac{TP+TN}{TP+TN+FP+FN}$$

11 $$P=\frac{TP}{TP+FP}$$

12 $$R=\frac{TP}{TP+FN}$$

13 $${F}_{1}=\frac{2PR}{P+R}$$

where TP, TN, FP and FN refer to true positives, true negatives, false positives and false negatives, respectively.

### Comparison with SOTA networks

We compare the SOTA model with DASUNet to verify the effectiveness of the model in this article. The comparison model is as follows***:

FC-EF^13^ uses early fusion for CD.

FC-Siam-Diff^13^ achieves CD by fusing the differential features of the Siamese network.

FC-Siam-Conc^13^ achieves CD by fusing bitemporal features of the Siamese network.

L-UNet^41^ uses a UNet-like structure to model encoder extraction features through an integrated fully convolutional LSTM block to achieve CD.

IFNet^12^ designs a depth-supervised differential discriminant network.

SNUNet^29^ combines the nested and densely connection with Siamese network, based on UNet++. To be fair, we choose SNUNet-24 with the same number of parameters size as DASUNet in this article.

USSFC-Net^20^ designs the multi-scale decoupled convolution and uses a non-weighted shared pseudo-Siamese structure to extract bitemporal features.

TinyCD

^28^ uses a pre-trained EfficientNet backbone to extract features, mix and attention mask block for feature information enhancement, and a pixel-by-pixel classifier to generate the final output.

ChangeFormer^22^ is a Transformer-based Siamese architecture that unifies a hierarchical transformer encoder with a multi-layer-aware decoder in a Siamese architecture.

IDET^33^ is an iterative differential enhancement transformer that consists of three transformers, two for extracting telematics from two images and one for enhancing feature differences. At the same time, the author uses it for change detection.

ScratchFormer^23^ uses a scrambled sparse attention operation to capture the intrinsic features of the CD data, and introduces a Change Detection Feature Fusion module to fuse features from input image pairs.

Swin-UNet-CD^30^ is an early fusion strategy for Swin-Unet change detection network, we only adjusted the number of input channels for Swin-Unet network.

DASUNet-32 is based on DASUNet-64 and the number of channels is halved.

### Comparison experiments

Table 1 show the results of the comparative experiments on the two datasets, respectively. On the CDD dataset, the F1 index of DASUNet is 0.85% higher than the current best network SNUNet-24. The F1 index of DASUNet is 0.36% higher than the current best network TinyCD on the WHU-CD dataset. It is worth mentioning that DASUNet-32 can still achieve good results on the two datasets, which is more balanced on both datasets than the USSFC-Net network.

**Table 1:** Comparison of experimental results on CDD and WHU-CD.

Method type	Network	CDD				WHU-CD			
		P (%)	R (%)	F1 (%)	OA (%)	P (%)	R (%)	F1 (%)	OA (%)
CNN	FC-EF	75.88	43.73	55.49	91.95	76.67	63.93	69.73	95.24
	FC-Siam-conc	74.94	50.21	60.13	92.36	41.16	85.22	55.51	88.29
	FC-Siam-diff	80.39	56.74	66.53	93.45	43.24	88.58	58.12	89.06
	L-UNet	91.79	81.35	86.25	97.02	64.54	78.29	70.76	94.45
	IFNet	92.35	84.58	88.29	97.43	*90.39*	87.02	88.67	98.09
	SNUNet-24	94.80	92.18	93.47	98.52	90.15	89.46	89.81	98.25
	USSFC-Net	91.29	83.16	87.04	97.16	89.49	90.31	89.90	98.26
	TinyCD	90.48	82.39	86.24	96.98	89.63	*90.40*	90.01	98.28
	DASUNet-32	92.93	89.38	91.12	98.01	89.81	86.24	87.98	97.98
	DASUNet-64	*94.94*	*93.7*	*94.32*	*98.71*	90.01	90.39	*90.37*	*98.33*
Transformer	ChangeFormer	90.11	77.39	83.36	96.43	88.04	80.66	84.19	97.40
	IDET	85.01	62.90	72.30	94.47	72.94	81.06	76.78	95.80
	ScratchFormer	91.17	80.67	85.60	96.88	88.77	83.45	86.03	97.68
	Swin-UNet-CD	81.96	59.48	68.93	93.85	87.39	73.08	79.59	96.79

The best two results are in italics and underlined, respectively.

**Figure 5:**
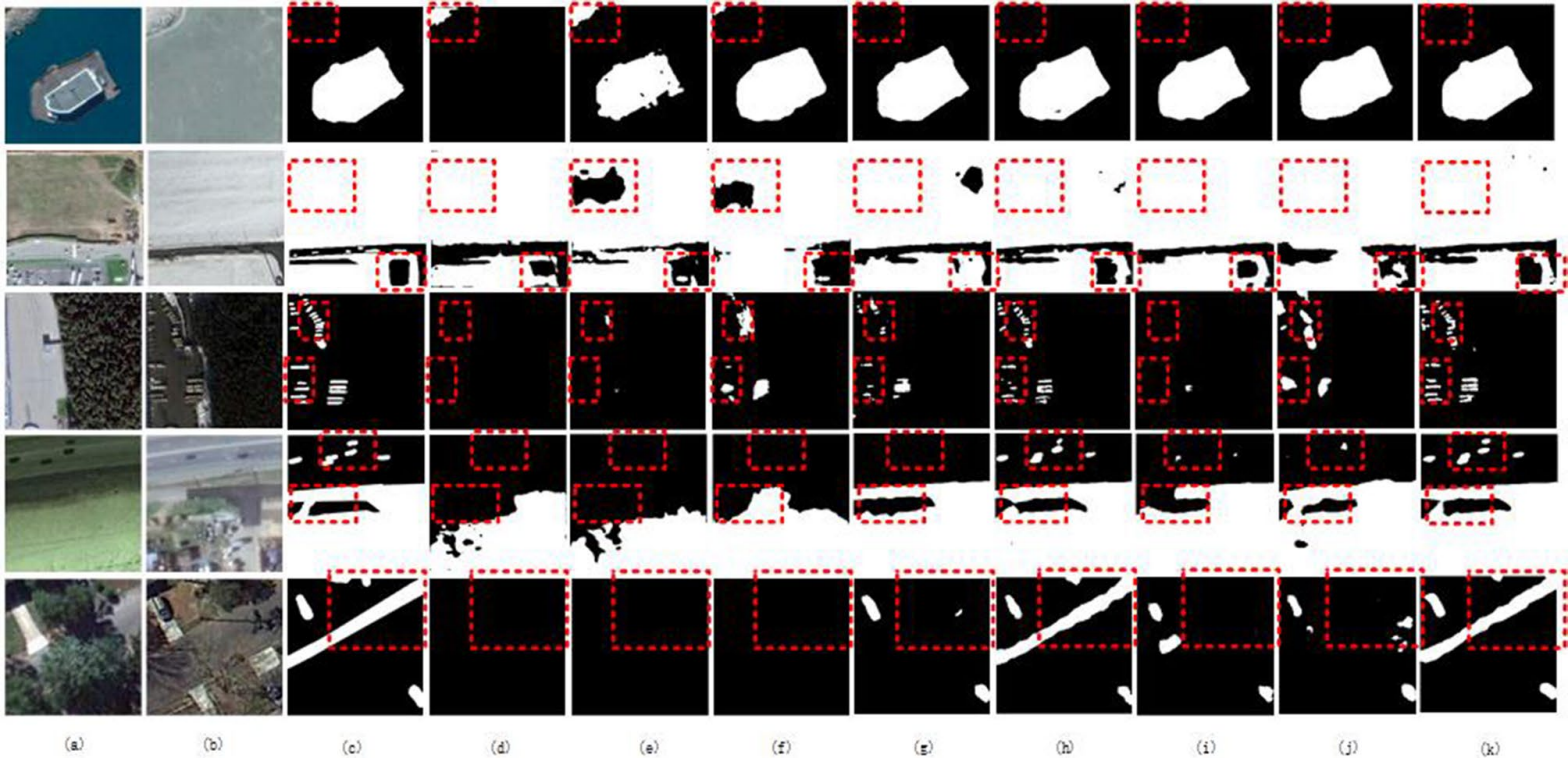
Visual comparison results on the CDD^38^; (**a**) Image at time1; (**b**) Image at time2; (**c**) Ground truth; (**d**) FC-EF; (**e**) FC-Siam-Conc; (**f**) FC-Siam-Diff; (**g**) IFNet; (**h**) SNUNet-24; (**i**) USSFC-Net; (**j**) ChangeFormer; and (**k**) DASUNet. The black area is the non-variation category, and the white area is the variation class.

Figure 5 shows the visual comparison results on the CDD dataset. In the first row of building detection, there are obvious false detections in the upper left corner of FC-EF, FC-Siam-Conc, and DASUNet and FC-Siam-Diff achieves good results in detecting complete large areas. In the second line of road detection, there are obvious missed detections of FC-Siam-Conc, IFNet and FC-Siam-Diff, and the change area predicted by DASUNet is relatively complete. In the detection of vehicles in line three, FC-Siam-Diff, ChangeFormer and IFNet have obvious regional connections, and the network in this article can clearly see the boundaries of each vehicle. In the detection of both large area and small target in the fourth row, the other networks did not detect the small vehicle targets, and there are serious false detections. But the proposed network in this article achieves the synchronous detection of large areas and small targets. In the 5th line of vehicle and road detection, due to the influence of the season, the leaves are obviously occluded, and the other networks do not detect continuous road information, and the network in this article has clear road boundary information.

**Figure 6:**
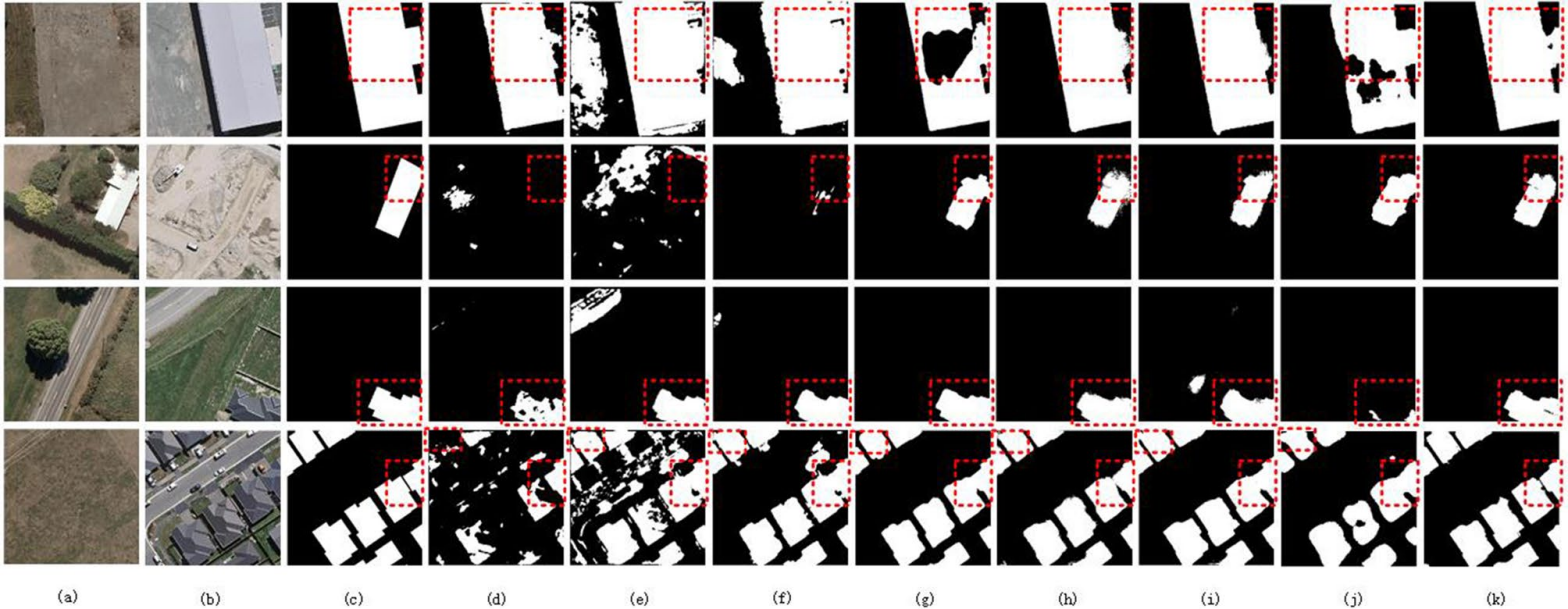
Visual comparison results on the WHU-CD^38^; (**a**) Image at time1; (**b**) Image at time2; (**c**) Ground truth; (**d**) FC-EF; (**e**) FC-Siam-Conc; (**f**) FC-Siam-Diff; (**g**) IFNet; (**h**) SNUNet-24; (**i**) USSFC-Net; (**j**) ChangeFormer; and (**k**) DASUNet. The black area is the non-variation category, and the white area is the variation class.

Figure 6 shows the visual comparison results on the WHU-CD dataset. In the detection of the first line of buildings, although the boundary information of the building is obvious, there are serious boundary misdetections in FC-Siam-Conc and FC-Siam-Diff, while there are obvious missed detections in IFNet, ChangeFormer and FC-EF. In the second line of building disappearance detection, the boundary of SNUNet-24 is blurred due to the occlusion of leaves, and the complete boundary information is detected by IFNet, USSFC-Net and the proposed network. In the third row, compared with IFNet, SNUNet-24 and USSFC-Net, DASUNet detect more complete building boundary information. At last, in the fourth row of building cluster detection, IFNet, SNUNet-24, ChangeFormer and USSFC-Net all have obvious boundary connections, and the boundaries of each building can be detected by DASUNet.

### Ablation experiments

In this section, ablation experiments were performed between the ASPP module and the DS module to evaluate the performance of each module. As can be seen from Table 2,

**Table 2:** Ablation experimental results on CDD and WHU-CD.

Network	CDD				WHU-CD			
	P (%)	R (%)	F1 (%)	OA (%)	P (%)	R (%)	F1 (%)	OA (%)
Base	94.30	91.58	92.92	98.40	88.55	86.72	87.63	97.90
Base + ASPP	94.83	*93.43*	*94.12*	*98.66*	*90.60*	88.01	89.28	98.19
Base + DS	**95.04**	92.86	93.93	98.62	**90.80**	*88.91*	*89.85*	*98.28*
Base + ASPP + DS	*94.94*	**93.70**	**94.32**	**98.71**	90.01	**90.39**	**90.37**	**98.33**

The best two results are in bold and italics, respectively.

F1 increases by 1.2% and 1.56% respectively after adding the ASPP module, indicating that the model extracts richer multi-scale features after adding the ASPP module, and F1 increases by 1.01% and 2.22% respectively after adding the deep supervision module, indicating that the added side auxiliary branches play a better role in the final prediction of semantic information at all levels. At the same time, in F1, the complete model with two modules is increased respectively by 1.4% and 2.74%, achieving a good module integration effect. It is worth noting that the indices of the complete model are more balanced, while the single-module model tends to focus on accuracy without fully considering the false positive and false negative rates, resulting in the F1 index being inferior to the complete model. This also reflects the better real performance of the complete model.

**Figure 7:**
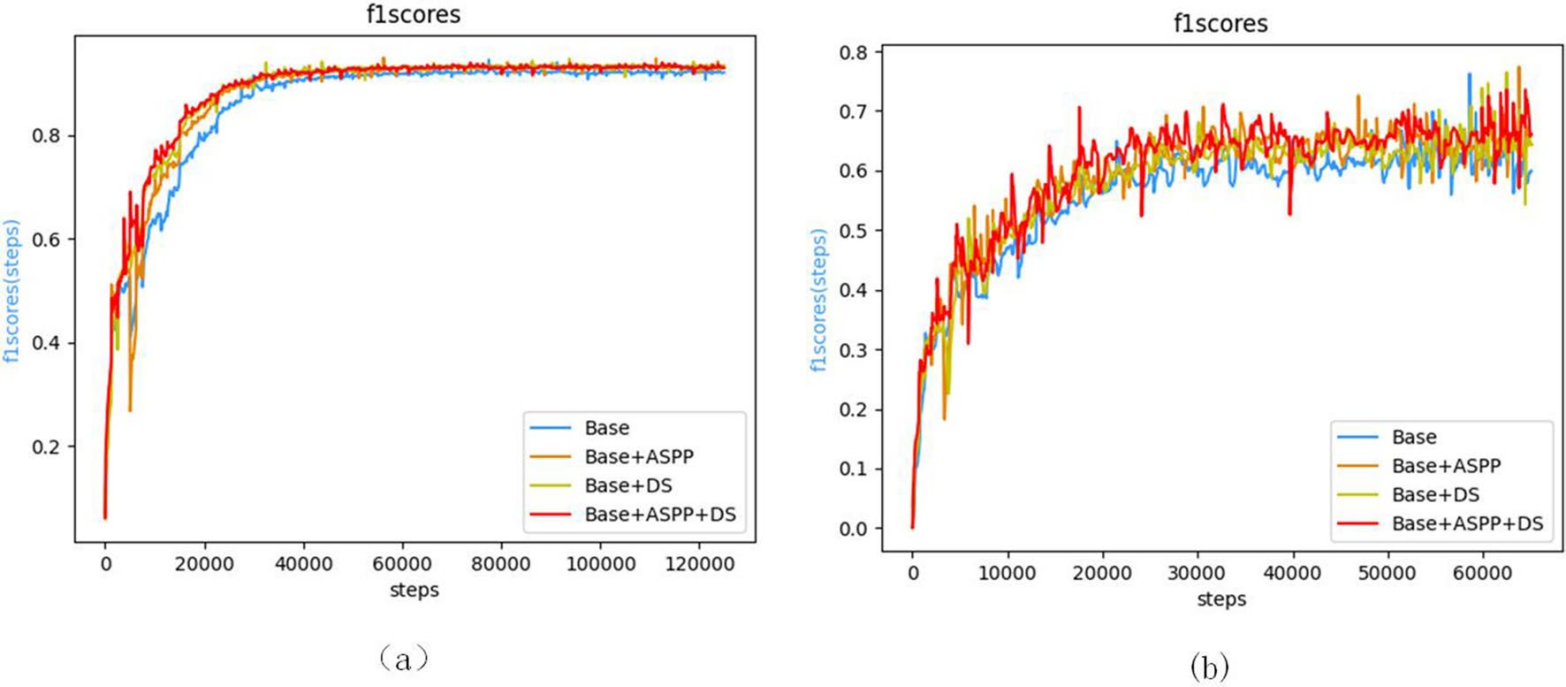
F_1_scores training curve (**a**) on CDD (**b**) and on WHU-CD.

Figure 7 show the training curves of F1 for each module in the ablation experiment, and the curve performance of each module is basically consistent with the data in Table 2 when the learning rate decay is consistent.

## Discussion

We verify the effectiveness of the proposed network on the CDD dataset and the WHU-CD dataset, respectively. Compared with other SOTA networks, such as TinyCD, which performs well on the building dataset but does not perform well on the seasonal change dataset CDD, DASUNet shows good performance in both the boundary information prediction of large targets and the shape information prediction of small targets. The key reason for the better performance of this network in CD is the introduction of ASPP blocks and deep supervision modules. From the analysis, it can be seen that ordinary convolutional blocks can usually only extract single-scale image features, so we use ASPP to replace the underlying convolutional blocks, so that the receptive field is expanded and multi-scale fusion features are obtained, which contains richer feature information and is more robust to seasonal changes and objects of different scales. In addition, the general training model lacks the supervision of the middle layer and does not pay enough attention to the effective layer information, so we use the DS module to supervise the hidden layer and fully explore the value of semantic graphs at different scales.

As can be seen from Table 3, the proposed model still leaves something to be desired. The network in this paper does not have an advantage in terms of the number of parameters and the amount of computation. It is worth noting that the CNNs-based CD model is better than the Transformer-based method in terms of the number of parameters and the amount of computation, but the results are opposite in terms of training and testing time, and both have their own advantages. Therefore, in the future, this paper plans to combine Transformer with CNN, and at the same time choose a more novel and sophisticated feature processing method to achieve better performance and control the difficulty of training and transplantation.

**Table 3:** Computational and parametric quantities comparison of experiment results on CDD and WHU-CD.

Method type	Network	Params(M)	Gflops(G)	CDD		WHU-CD	
				Train_Epoch(S)	Test_Epoch(S)	Train_Epoch(S)	Test_Epoch(S)
CNN	FC-EF	*1.35*	*3.58*	*578.95*	**61.62**	354.50	18.25
	FC-Siam-conc	1.55	5.33	676.39	*65.77*	*351.09*	*17.52*
	FC-Siam-diff	*1.35*	4.73	685.02	70.45	356.32	**17.25**
	L-UNet	8.45	17.33	870.42	98.04	443.59	23.78
	IFNet	35.99	82.27	893.78	115.45	450.91	28.96
	SNUNet-24	6.77	30.90	873.24	103.89	470.66	26.31
	USSFC-Net	1.52	4.86	830.91	90.23	430.95	23.44
	TinyCD	**0.29**	**1.54**	704.65	80.57	329.30	20.92
	DASUNet-32	2.27	25.61	1022.98	135.88	515.98	34.12
	DASUNet-64	9.07	100.93	1731.19	226.62	874.34	56.94
Transformer	ChangeFormer	29.84	11.65	688.44	77.17	347.97	17.81
	IDET	45.09	124.19	1232.82	126.14	669.25	32.28
	ScratchFormer	36.92	196.59	2690.59	291.41	1387.35	71.66
	Swin-UNet-CD	27.15	7.75	**534.23**	70.77	**316.49**	18.63

The best two results are in bold and italics, respectively.

Train_Epoch indicates the training time per epoch, and Test_Epoch indicates the time for each epoch of testing.

## Conclusions

In the article, we propose a CD network for high-resolution remote sensing images, which adopts an end-to-end approach and directly learns the features of the dataset without the help of transfer learning. The network adopts a Siamese architecture, which integrates the global feature information through full-scale skip connection structure, and realizes end-to-end training. At the same time, the network uses ASPP module in the coding stage and the deep supervision mechanism in the decoding stage, which integrates the change characteristics of multiple scales and makes use of the role of feature information of each scale in the final prediction. Through experimental comparison and visualization results, the proposed network has achieved competitive performance on the public dataset CDD and WHU-CD. In F1, it increased by 0.85% and 0.36%, respectively.

There are still many shortcomings in the network of this article. In the future research, we will explore the method of using transformer to process multi-scale features to further improve the fineness of boundary detection. At the same time, through the adjustment of the model, we plan to apply the proposed method to more remote sensing image change detection scenarios such as multi-category extraction and road detection.

## Data Availability

The datasets in this article are public. The CDD dataset can be downloaded from the https://drive.google.com/file/d/1GX656JqqOyBi_Ef0w65kDGVto-nHrNs9, and the WHU-CD dataset can be downloaded from the http://gpcv.whu.edu.cn/data/building_dataset.html.
